# Imatinib with intensive chemotherapy in AML with t(9;22)(q34.1;q11.2)/BCR::ABL1. A DATAML registry study

**DOI:** 10.1038/s41408-024-01069-9

**Published:** 2024-05-31

**Authors:** Camille Gondran, Pierre-Yves Dumas, Emilie Bérard, Audrey Bidet, Eric Delabesse, Suzanne Tavitian, Thibaut Leguay, Françoise Huguet, Cécile Borel, Edouard Forcade, François Vergez, Jean-Philippe Vial, Jean Baptiste Rieu, Nicolas Lechevalier, Isabelle Luquet, Alban Canali, Emilie Klein, Audrey Sarry, Anne-Charlotte de Grande, Arnaud Pigneux, Christian Récher, Laetitia Largeaud, Sarah Bertoli

**Affiliations:** 1 Service d’Hématologie, Centre Hospitalier Universitaire de Toulouse, Institut Universitaire du Cancer de Toulouse Oncopole, Toulouse, France; 2 Service d’Hématologie Clinique et de Thérapie Cellulaire, Centre Hospitalier Universitaire de Bordeaux, F-33000 Bordeaux, France; 3 Université de Bordeaux, 33076 Bordeaux, France; 4 Institut National de la Santé et de la Recherche Médicale, U1035, 33000 Bordeaux, France; 5 Service d’Epidémiologie, Centre Hospitalier Universitaire de Toulouse, CERPOP, Inserm, Toulouse, France; 6 Université Toulouse III Paul Sabatier, Toulouse, France; 7 Laboratoire d’Hématologie Biologique, Centre Hospitalier Universitaire de Bordeaux, F-33000 Bordeaux, France; 8 Laboratoire d’Hématologie Biologique, Centre Hospitalier Universitaire de Toulouse, Institut Universitaire du Cancer de Toulouse Oncopole, Toulouse, France

**Keywords:** Acute myeloid leukaemia, Cancer genetics

## Abstract

Acute myeloid leukemia (AML) with t(9;22) (q34.1; q11.2)/*BCR::ABL1*, a distinct entity within the group of AML with defining genetic abnormalities, belong to the adverse-risk group of the 2022 ELN classification. However, there is little data on outcome since the era of tyrosine kinase inhibitors. Among 5819 AML cases included in the DATAML registry, 20 patients with de novo *BCR::ABL1*^+^AML (0.3%) were identified. Eighteen patients treated with standard induction chemotherapy were analyzed in this study. Imatinib was added to chemotherapy in 16 patients. The female-to-male ratio was 1.25 and median age was 54 years. The t(9;22) translocation was the sole chromosomal abnormality in 12 patients. Main gene mutations detected by NGS were *ASXL1*, *RUNX1* and *NPM1*. Compared with patients with myeloid blast phase of chronic myeloid leukemia (CML-BP), de novo *BCR::ABL1*^+^AML had higher WBC, fewer additional chromosomal abnormalities, lower CD36 or CD7 expression and no *ABL1* mutations. Seventeen patients (94.4%) achieved complete remission (CR) or CR with incomplete hematologic recovery. Twelve patients were allografted in first remission. With a median follow-up of 6.3 years, the median OS was not reached and 2-year OS was 77% (95% CI: 50–91). Four out of five patients who were not transplanted did not relapse. Comparison of *BCR::ABL1*^+^AML, CML-BP, 2017 ELN intermediate (*n* = 643) and adverse-risk patients (*n* = 863) showed that patients with *BCR::ABL1*^+^AML had a significant better outcome than intermediate and adverse-risk patients. *BCR::ABL1*^+^AML patients treated with imatinib and intensive chemotherapy should not be included in the adverse-risk group of current AML classifications.

## Introduction

Acute myeloid leukemia (AML) with t(9;22) (q34.1; q11.2)/*BCR::ABL1* is now considered as a distinct entity within the group of AML with defining genetic abnormalities in both the World Health Organization Classification of Haematolymphoid Tumours (WHO 2022) and the International Consensus Classification of Myeloid Neoplasms and Acute Leukemias [1, 2]. This is a very rare entity with an estimated prevalence of 0.1–3% of AML cases [3–6]. Contrary to other genetically defined AML, AML with *BCR::ABL1* still requires ≥20% blasts for diagnosis to avoid potential overlap with accelerated phase of chronic myeloid leukemia (CML-AP). Indeed, the distinction of de novo AML with *BCR::ABL1* from initial myeloid blast phase of CML (CML-BP) can be challenging although patient medical history and a few biological characteristics may help to differentiate them. CML-BP are characterized by frequent splenomegaly, significant blood or marrow basophilia, additional chromosomal abnormalities (ACA), or *ABL1* mutations [3, 5, 7, 8]. AML with *BCR::ABL1* is much less described but has been associated with a lower frequency of ACA and with a unique gene signature including deletion in *IKZF1*, *CDKN2A* and/or in the immunoglobulin and T cell receptor genes [9]. *CD25* and *ID4* mRNA expression might also differentiate AML with *BCR::ABL1* from CML-BP [10]. Recent molecular data revealed that, opposite to CML-BP, AML with *BCR::ABL1* can be associated with *NPM1* mutations while no *ABL1* mutations have been described to date [3, 5]. However, other studies failed to detect *NPM1* mutations in AML with *BCR::ABL1* [7, 8].

AML with *BCR::ABL1* belong to the adverse risk group of the 2022 ELN classification [11]. In the United Kingdom Medical Research Council study on 5876 younger adult patients treated with intensive chemotherapy, the 47 patients with t(9;22) (q34.1; q11.2) AML had an overall survival (OS) of 11% and the t(9;22) was an independent predictor of poor outcome [4]. However, these results were drawn from earlier studies with heterogeneous treatment regimens including or not the use of tyrosine kinase inhibitors (TKIs). AML with *BCR::ABL1* is generally an exclusion criteria in clinical trials and only very large registry offer the opportunity to capture a sufficient number of patients to provide clinical insights. Thus, there is little data on outcome especially since the era of TKIs. In this study, we sought to update clinical presentation, results of treatments and outcome of patients with de novo AML with t(9;22)(q34.1;q11.2)/*BCR::ABL1*.

## Methods

### Patients

All patients aged ≥ 18 years with t(9;22) (q34.1; q11.2)/*BCR::ABL1* documented by karyotype or FISH and ≥ 20% blasts included in the DATAML registry between 2000 and 2021 were analyzed. The DATAML registry contains all patients with ≥ 20% blasts of myeloid lineage in the bone marrow or blood or with an AML diagnosis according to WHO classifications. De novo *BCR::ABL1*^+^ AML was defined as no previous history of CML, no previous treatment with TKI and ≥ 20% blasts in bone marrow. CML-BP was defined as the occurrence of ≥20% myeloid blasts in patients with previous diagnosis of CML in chronic phase. Patients with lymphoid CML-BP or with mixed-phenotype acute leukemia are not registered in DATAML. Additional data were retrospectively collected for this study including CML history (date of diagnosis, SOKAL and ELTS score, treatments), BCR-ABL1 isotype (P190, P210), *ABL1* mutations, anthracycline dose and TKIs during intensive chemotherapy, measurable residual disease (MRD) evaluations following induction, first consolidation cycle, before allogeneic hematopoietic cell transplantation (alloHCT), end of treatment or 100 days post alloHCT. Response to treatment, relapse, relapse-free survival (RFS), event-free survival (EFS), and OS were defined according to the ELN criteria [11]. This study was performed in accordance with the Declaration of Helsinki. DATAML was approved by French authorities and informed consent was provided to all patients.

### Cytogenetics

Conventional karyotyping was performed on the BM diagnostic aspirate after short-term culture (24–72 h). The chromosomes were analyzed after R and/or G-banding. All karyotypes were reported according to the the International System for Human Cytogenetic Nomenclature (ISCN 2020).

### Immunophenotyping

Multi-parameter flow cytometry (MFC) was performed on whole bone marrow (BM) or blood specimens using a standard stain-lyse-wash procedure with ammonium chloride lysis. 1 × 10^5^ cells were stained per analysis tube, and data were acquired on at least 1 × 10^4^ blasts when specimen quality permitted. Data on standardized 10-color staining combinations were acquired on Navios instruments analyzed using Kaluza (Beckman-Coulter). Several different tube configurations were used for leukemic bulk analysis, associated 20 different markers (CD3, CD7, CD11b, CD13, CD14, CD16, CD19, CD33, CD34, CD36, CD38, CD45, CD45RA, CD56, CD64, CD79a, CD117, CD133, HLA-DR, and MPO). A blast gate including CD45 dim mononuclear cells was analyzed according to cytomorphologic data. Leukemic hematopoietic stem and progenitor subpopulations were gated on CD34^+^ cells and selected according to their expression of CD38, CD45RA, CD135 and CD133. Flow cytometry-based immunophenotypic classification defining six stages of leukemia differentiation-arrest categories was based on CD34, CD117, CD13, CD33, MPO and HLA-DR expression [12]. For phenotypic comparisons, CML-BP (*n* = 9), *BCR::ABL1*^+^AML (*n* = 10) and non-*BCR::ABL1*^+^AML (*n* = 2455) were used.

### Next generation sequencing

Genomic DNA from bone marrow or blood samples was extracted by standard procedures and sequenced using an Illumina NextSeq550Dx (Bordeaux) or NextSeq500 (Toulouse) sequencers and Magnis SureSelect XT HS capture panel (Agilent, Santa Clara, CA, USA) covering the complete coding regions (and −2 to +2 splicing sites) of 49 genes recurrently mutated in myeloid neoplasms (*ANKRD26, ASXL1, ASXL2,BCOR, BCORL1, CALR, CBL, CCND2, CEBPA, CSF3R, CUX1, DDX41, DHX15, DNMT3A, ETNK1, ETV6, EZH2, FLT3, GATA1, GATA2, GNAS, GNB1,IDH1, IDH2, IKZF1, JAK2, KDM6A, KIT, KRAS, MPL, MYC,NFE2, NPM1, NRAS, PHF6, PPM1D, PTEN, PTPN11, RAD21, RIT1, RUNX1, SAMD9, SAMD9L, SETBP1, SF3B1, SH2B3, SMC1A, SMC3, SRSF2, STAG2, TET2, TERC, TERT, TP53, U2AF1, UBA1, WT1 and ZRSR2*). Raw NGS data were analyzed using different “variant callers”: Mutect2, Varscan2, Lofreq and Vardict (Bordeaux) or MuTect2, HaplotypeCaller (both from the GATK suite developed by the Broad Institute) and SureCall (Agilent) algorithms (Toulouse) for variant calling aggregated in the in-house remote pipeline for data visualization, elimination of sequencing/mapping errors and retention of variants with high quality metrics. Variant interpretation was performed considering minor allele frequencies (MAF) in the public GnomAD database of polymorphisms (variants with MAF > 0.02 in overall population/global ancestry or sub-continental ancestry are excluded), variant allele frequencies (VAF), prevalence and clinical interpretation (COSMIC, protein impact). All variants were checked manually on IGV and named according to the Human Genome Variation Society.

### Measurable residual disease

RNA was extracted from blood or bone marrow cells. Quantification of *BCR::ABL1* transcript levels was performed after reverse transcription and quantitative real-time polymerase chain reaction (RT-qPCR) according to the Europe against cancer (EAC) protocol using ABL1 as control gene [13]. Results are expressed as a percentage of *BCR::ABL1/ABL1*.

### Statistical analysis

Data analysis was performed using Stata software (Statistical Software: Release 18.0. Stata Corporation, College Station, Texas, USA). All reported *P*-values were two-sided and the significance threshold was set at <0.05. Comparisons of the patients’ characteristics between groups (de novo *BCR::ABL1*^+^AML vs CML-BP) were assessed using Student’s *t* test (or the Mann–Whitney test if necessary) for continuous variables, and the chi2-test (or Fisher’s exact test if necessary) for categorical variables (including response to induction). For survival endpoints (EFS, RFS and OS), Kaplan–Meier survival curves were drawn and described using median in months (with IQR) as well as survival rates at 2 and 5 years. Differences in survival functions were tested using the log-rank test. The median follow-up (and its interquartile range (IQR)) was described by the reverse Kaplan–Meier technique. In the flow cytometry analysis, comparisons were performed using a Mann–Whitney test for continuous variables and Fisher’s exact test for categorical variables with GraphPad Prism. Ward’s clustering and PCA were conducted with Tanagra statistical software. Statistical test results are graphically expressed: *p < 0.05, **p < 0.01, ***p < 0.001.

## Results

### Study population, karyotype and NGS

**Fig. 1:**
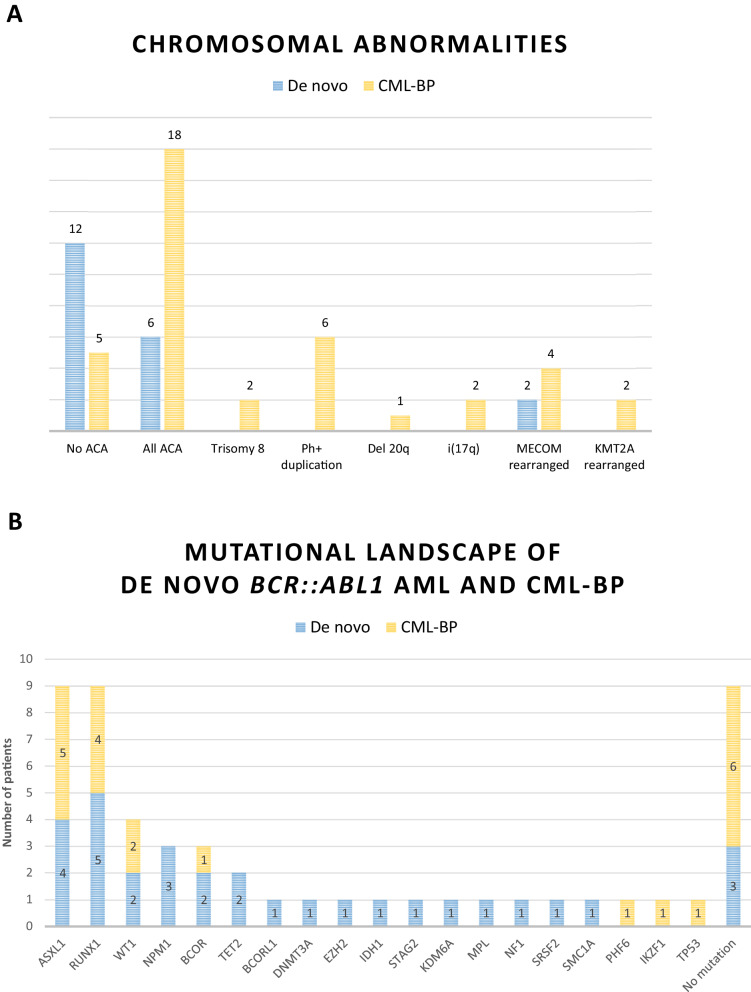
Chromosomal abnormalities and mutations. Additional chromosomal abnormalities (**A**) and mutational profile (**B**) in patients with de novo *BCR::ABL1*^+^AML or CML-BP. **A** de novo *BCR::ABL1*^+^AML in blue (*n* = 20), CML-BP in yellow (*n* = 29).

Forty-nine patients with t(9;22) (q34.1; q11.2)/*BCR::ABL1* among 5819 AML cases included in the DATAML registry between 2000 and 2021 were identified including 20 patients with de novo *BCR::ABL1*^+^AML (0.3%). Detailed individual characteristics of each patient are presented in Supplementary Table 1. The t(9;22) translocation was the sole chromosomal abnormality in 12 patients with de novo *BCR::ABL1*^+^AML and all karyotypes showed at least 80% Philadelphia chromosome-positive mitoses (Fig. 1A, Supplementary Table 1). De novo *BCR::ABL1*^+^  AML rarely showed the ACA usually observed in CML-AP or CML-BP. There was no duplication of the Philadelphia chromosome (vs. 6/23 in CML-BP), no isochromosome 17q (vs. 2/23 CML-BP) and only 2/18 cases had a *MECOM* rearrangement (vs. 5/23 *MECOMr* in CML-BP). NGS analyses were performed on available banked samples regardless of treatment. Thirty-two mutations were detected in 15 patients with de novo *BCR::ABL1*^+^ AML whereas 21 mutations were detected in 19 CML-BP patients. The median number of mutations detected in 17 patients with de novo *BCR::ABL1*^+^ AML was 2 (IQR, 1–3) *vs*. 1 (IQR, 0–2) in 19 CML-BP patients (*p* = 0.108). Main gene mutations in de novo *BCR::ABL1*^+^ AML *were RUNX1* (*n* = 5), *ASXL1* (*n* = 4), *NPM1* (*n* = 3), *BCOR* (*n* = 2), *TET2* (*n* = 2) and WT1 (*n* = 2) (Fig. 1B) and no patients had detectable *ABL1* mutations.

Five patients with CML-BP and two with de novo *BCR::ABL1*^+^AML who did not receive intensive chemotherapy were excluded from the analysis of treatment response and outcome. The characteristics of the 18 de novo *BCR::ABL1*^+^AML and 24 CML-BP patients who received intensive chemotherapy are shown in Table 1. For CML-BP patients, details of previous CML prognostic scores and treatment are shown in Supplementary Table 2. In the de novo *BCR::ABL1*^+^AML group, the female-to-male ratio was 1.25 and median age was 54 years. Extra medullary disease was documented in eight patients including splenomegaly by physical examination in six, and the median white blood cell count was 91.5 G/L.

**Table 1:** Patients characteristics.

	All patients *N* = 42	De *novo BCR::ABL1* AML *N* = 18	CML-BP *N* = 24	*p*-value
Age (years), median (min-max)	52.3 (22–71)	54.1 (22–71)	52.3 (29–66)	0.765
Sex *n* (%) Male Female	19 (45) 23 (55)	8 (44) 10 (56)	11 (46) 13 (54)	0.929
Performance status, *n* (%)				0.839
0	9 (23.7)	4 (25)	5 (22.7)	
1	17 (44.7)	8 (50)	9 (40.9)	
≥ 2	12 (31.6)	4 (25)	8 (36.4)	
Extramedullary disease, *n* (%)	21 (52.5)	8 (47.1)	13 (56.5)	0.554
Hepatomegaly	8 (20)	4 (23.5)	4 (17.4)	0.702
Splenomegaly	17 (42.5)	6 (35.3)	11 (47.8)	0.428
Polyadenopathy	5 (12.5)	2 (11.8)	3 (13)	1
Chloroma	1 (2.5)	0 (0)	1 (4.3)	1
CNS	1 (2.5)	0 (0)	1 (4.3)	1
Leukostasis, *n* (%)	3 (7.2)	0 (0)	3 (12.5)	0.497
Hemoglobin (g/dL), median (IQR)	10.3 (8.5–11.5)	10.9 (7.8–11.7)	10.1 (8.9–11.2)	0.740
WBC (Giga/L), median (IQR)	42.3 (14–107.8)	91.5 (20.8–143)	33 (6.3–74.95)	0.079
ANC (Giga/L), median (IQR)	7.3 (2.04–30.91)	11.20 (3.92–33.23)	4.7 (1.39–13.41)	0.177
Platelets (Giga/L), median (IQR)	108.5 (68–256)	164.5 (53–256)	99.5 (74.5–249)	0.799
Blood blasts (%), median (IQR)	23.5 (9–56)	33.5 (18–63)	20.5 (7–50)	0.371
Bone marrow blasts (%), median (IQR)	35 (24–65)	32 (21–71)	38 (28–57)	0.605
*BCR::ABL1* isotype, *n* (%)				1
p210	32 (76)	14 (77.8)	18 (75.0)	
p190	4 (9.5)	2 (11.1)	2 (8.3)	
Unknown	6 (14.5)	2 (11.1)	4 (16.7)	
*ABL1* mutation (*n* = 14), *n* (%)				0.209
Yes	3 (21.4)	0	3 (37.5)	
No	11 (78.6)	6 (100)	5 (62.5)	
LDH (UI/L), median (IQR)	929 (528–1707)	799 (692–1512)	969 (426–1707)	0.777
Creatinine (µmol/L), median (IQR)	79.0 (66.0–89.0)	81 (73.0–97.0)	75.5 (64.0–89.0)	0.476
Bilirubin (µmol/L), median (IQR)	9.5 (7.0–13.2)	10.1 (6.9–13.0)	9.0 (7.1–13.4)	0.795
Albumin (g/L), median (IQR)	38.0 (33.3–42.0)	38.1 (33.3–43.0)	38.0 (33.7–41.0)	0.486
Ferritin (µg/L), median (IQR)	384.0 (130.0–832.0)	344.5 (130.4–484.5)	628.8 (109.0–1661.0)	0.324

*CML-BP* CML-blast phase, *IQR* Inter-Quartile Range, *Min* Minimum, *Max* Maximum, *CNS* central nervous system, *WBC* white blood cell count, *ANC* absolute neutrophil count, *LDH* lactate dehydrogenase.

### Immunophenotyping

**Fig. 2:**
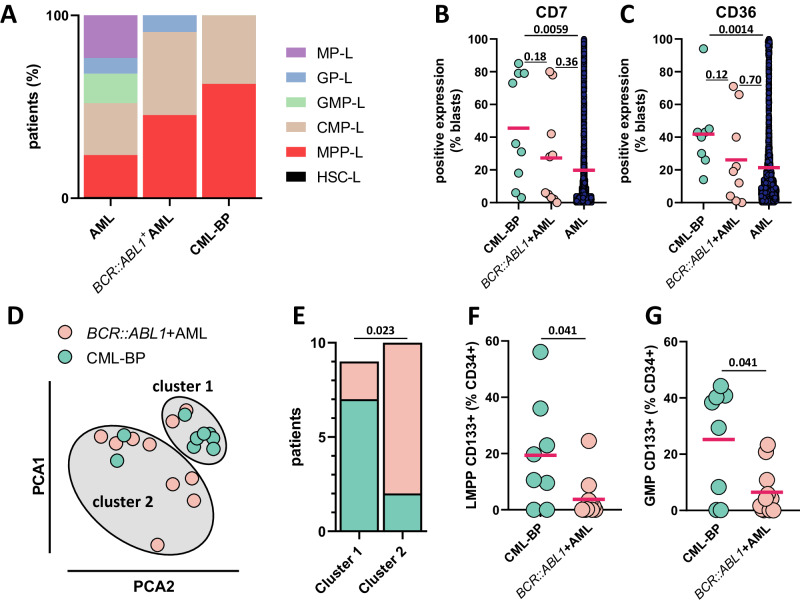
Immunophenotyping. **A** Immunophenotypic classification: HSC-L hematopoietic stem cells-like, MPP-L multipotent progenitors-like, CMP-L common myeloid progenitors-like, GMP-L granulocyte–monocyte progenitors, GP-L granulocyte progenitors-like, MP-L monocyte progenitors-like. **B** CD7 expression in CML-BP, de novo *BCR::ABL1*^+^AML or *BCR::ABL1* negative AML. **C** CD36 expression in CML-BP, de novo *BCR::ABL1*^+^AML or *BCR::ABL1* negative AML (AML). **D**, **E** Hierarchical clustering based on frequencies of hematopoietic stem and progenitor subpopulations. **F**, **G** CD133^+^ LMPP and CD133^+^ GMP frequencies in CML-BP and de novo *BCR::ABL1*^+^AML.

AML are phenotypically stratified based on stage of leukemia arrest, which characterizes the differentiation block in the human hematopoietic hierarchy [12]. We investigated the phenotype of 11 de novo *BCR::ABL1*^+^AML and 8 CML-BP. Both groups exhibited a significant enrichment in multipotent progenitor-like phenotype (MPP-L, odds ratio 4.83, *p* = 0.008 and 5.42, *p* = 0.0005, respectively) compared with a series of 2230 patients with *BCR::ABL1* negative AML (Fig. 2A and Supplementary Fig. 1). Among the 20 myeloid and lymphoid markers analyzed, higher expression levels of CD7 and CD36 were observed in CML-BP compared with *BCR::ABL1* negative AML (36% vs 6%, *p* = 0.0059 and 41% vs 11%, *p* = 0.0014, respectively; Fig. 2B, C). *BCR::ABL1*^+^AML showed intermediary level of expression of CD7 and CD36 without statistical difference with the other groups. Unsupervised hierarchical clustering was used to group patients based on their frequencies of hematopoietic stem and progenitor subpopulations measured by flow cytometry and normalized to the proportion of the CD34^+^ compartment. A dendrogram built using Ward’s clustering method identified two clusters (Fig. 2D). Cluster 1 was enriched in CML-BP whereas cluster 2 was enriched in de novo *BCR::ABL1*^+^AML (Fig. 2E). Principal component analysis was then used to classify the main sources of variation among patients. Specifically, frequencies of CD133^+^ lymphoid-primed multipotent progenitors (LMPP) and CD133^+^ granulocyte–monocyte progenitors (GMP) were significantly higher in CML-BP compared to de novo *BCR::ABL1*^+^AML (Fig. 2F, G).

### Treatment and response

All patients with de novo *BCR::ABL1*^+^AML received induction chemotherapy mainly with daunorubicin (60–90 mg/m², 3 days) and cytarabine (100–200 mg/m², 7 days) (Table 2). Imatinib was added from day 1 or day 8 of induction in 16 patients. The daily dose of imatinib was 400 mg, 600 mg or 800 mg in 1, 10 and 5 patients, respectively. The remaining two patients only received induction chemotherapy before imatinib was available. Seventeen patients (94.4%) achieved complete remission (CR, *n* = 11) or CR with incomplete hematologic recovery (CRi, *n* = 6). Only one patient needed a second cycle to reach CR. There was no early death during induction. Consolidation chemotherapy was imatinib in combination with intermediate to high-dose cytarabine in 10 patients or mini-consolidation in 5 patients whereas 2 patients proceeded directly to allogeneic stem-cell transplantation (Table 2). Four patients switched to a second generation TKI (dasatinib, *n* = 3; ponatinib, *n* = 1) because of imatinib-induced adverse events. Twelve patients (67%) were allografted in first remission with myeloablative (*n* = 7) or reduced-intensity (*n* = 5) conditioning (Table 2). Five patients received maintenance with imatinib or nilotinib. Two patients had maintenance therapy after allogeneic stem cell transplantation (one for six years and still ongoing, one for four years before dying of metastatic lung cancer). Three patients who were not transplanted had maintenance therapy (two still ongoing after four and seven years of maintenance and one had a 4 year maintenance therapy before discontinuing it and is still alive and disease-free two years later). Of the 12 patients who were informative for MRD evaluation in blood and/or bone marrow, four and six patients had *BCR::ABL1* transcript <0.1% after induction and end of consolidation/pre-alloHCT, respectively. Only two patients had undetectable MRD after consolidation chemotherapy whereas eight patients had undetectable MRD after allogeneic stem cell transplantation. Of note, the CR/CRi rate was 79.2% in patients with CML-BP. Of the 12 CML-BP patients who were informative for MRD evaluation in blood and/or bone marrow, 4 and 6 patients had *BCR::ABL1* transcript <0.1% after induction and end of consolidation/pre-alloHCT, respectively. Seven patients had undetectable MRD after allogeneic stem cell transplantation.

**Table 2:** Treatment, response to induction chemotherapy and post remission therapy.

	All patients *N* = 42	*De novo BCR::ABL1* AML *N* = 18	CML-BP *N* = 24	*p*-value
Anthracycline, *n* (%)				0.561
Daunorubicin 45 mg/m² ×3d	6 (14.3)	3 (16.7)	3 (12.5)	
Daunorubicin 60 mg/m² ×3d^a^	21 (50.0)	6 (33.3)	15 (62.5)	
Daunorubicin 90 mg/m² ×3d	10 (23.8)	6 (33.3)	4 (16.7)	
Idarubicin 8 mg/m² ×5d	3 (7.1)	2 (11.1)	1 (4.2)	
Other^b^	2 (4.8)	1 (5.6)	1 (4.2)	
Tyrosine kinase inhibitors, *n* (%)				0.001
None	6 (14.3)	2 (11.1)	4 (16.7)	
Imatinib	24 (57.1)	16 (88.9)	8 (33.3)	
Nilotinib	1 (2.4)	0	1 (4.2)	
Dasatinib	7 (16.7)	0	7 (29.2)	
Ponatinib	4 (9.5)	0	4 (16.7)	
Response to induction (CR + CRi), *n* (%)	36 (85.7)	17 (94.4)	19 (79.2)	0.214
CR	23 (54.8)	11 (61.1)	12 (50)	0.474
CRi	12 (28.6)	6 (33.3)	7 (29.2)	0.921
Death during induction, *n* (%)	2 (4.8)	0	2 (8.3)	0.497
Consolidation with IDAC/HDAC^c^, *n* (%)	20 (47.6)	10 (55.6)	10 (41.7)	0.372
Number of cycles				
1	10 (50)	2 (20)	8 (80)	0.029
2	6 (30)	5 (50)	1 (10)	
3	4 (20)	3 (30)	1 (10)	
Cytarabine dose				
≤1.5 g/m^2^	6 (30)	2 (20)	4 (40)	0.628
>1.5 g/m^2^	14 (70)	8 (80)	6 (60)	
Mini-consolidation^d^, *n* (%)	7 (16.7)	5 (27.8)	2 (8.3)	0.118
TKI switch during first line treatment, *n* (%)	5/36 (13.2)	4/16 (25)	1/20 (5)	0.141
Maintenance therapy with TKI, *n* = 34 (%)	12/34 (35.3)	5/16 (31.3)	7/18 (38.9)	0.642
Imatinib	8 (66.6)	4 (80.0)	4 (57.1)	
Dasatinib	0	0	0	
Nilotinib	1 (8.3)	1 (20.0)	0	
Ponatinib	3 (25.0)	0	3 (42.9)	
AlloSCT, *n* (%)	27 (64.3)	12 (66.7)	15 (62.5)	0.779
Stem cell source				
Peripheral blood	18 (66.7)	8 (66.7)	10 (66.7)	0.666
Bone marrow	8 (29.6)	3 (25)	5 (33.3)	
Umbilical cord blood	1 (3.7)	1 (8.3)	0	
Donor				
HLA-matched sibling	8 (29.6)	4 (33.3)	4 (26.7)	0.526
Unrelated donor	18 (66.7)	7 (58.3)	11 (73.3)	
Haploidentical	1 (3.7)	1 (8.3)	0	
Conditioning				
MAC	14 (51.9)	7 (58.3)	7 (46.6)	1
RIC	12 (44.4)	5 (41.7)	7 (46.7)	
Sequential	1 (3.7)	0	1 (6.7)	

*CML-BP* CML-blast phase, *CR* complete remission, *CRi* CR with incomplete hematologic recovery, *IDAC* Intermediate dose cytarabine, *HDAC* high dose cytarabine, *TKI* tyrosine kinase inhibitor, *AlloSCT* allogeneic stem cell transplantation, *MAC* myeloablative conditioning, *RIC* reduced-intensity conditioning.

^a^One CML-BP patient only received 2 days of daunorubicin.

^b^One de novo *BCR::ABL1* patient received daunorubicin 30 mg/m² 5 days; one CML-BP patient received mitoxantrone + cytarabine + thioguanine.

^c^Two patients proceeded directly to transplantation.

^d^Mini-consolidation: idarubicin 8 mg/m² day 1 or daunorubicin 45 mg/m² day 1, cytarabine 50 mg/m²/12 h subcutaneously d1-5.

### Outcome

**Fig. 3:**
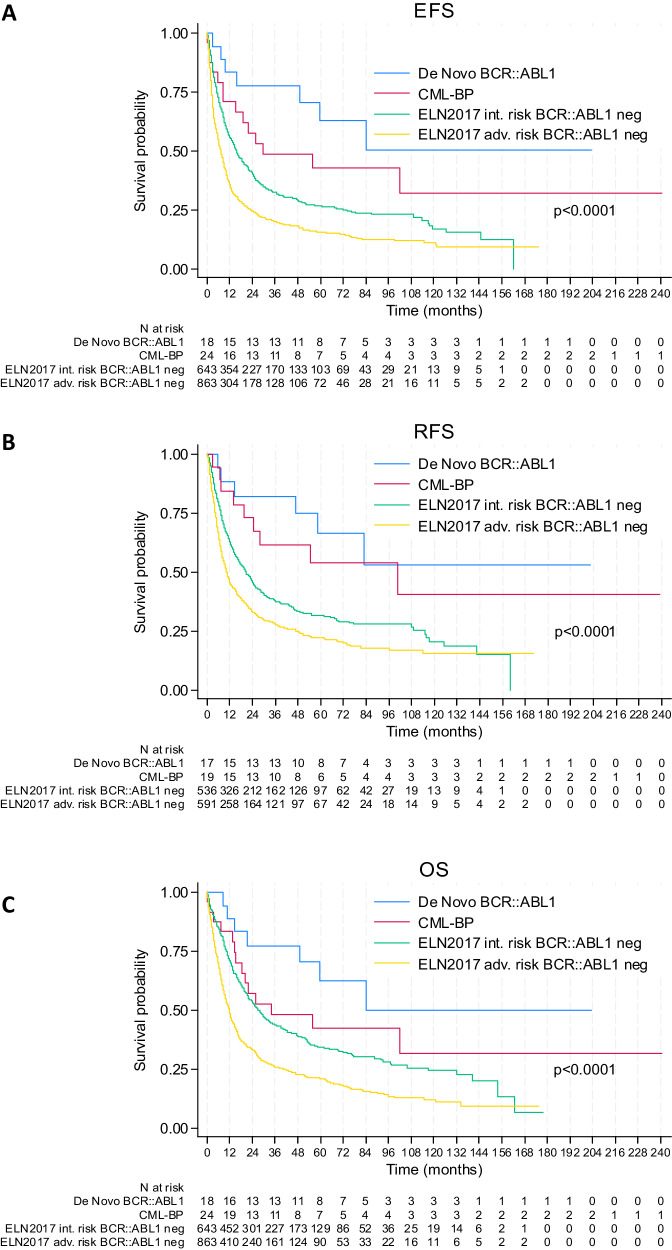
Survival curves in patients with de novo *BCR::ABL1*^*+*^AML, CML-BP, ELN 2017 intermediate or high-risk patients. **A** Event-free survival **(**EFS). **B** Relapse-free survival (RFS). **C** Overall survival (OS).

The median follow-up was 6.3 years (IQR 4.0–11.8). The median EFS of patients with *BCR::ABL1*^+^AML was not reached (IQR 49 months–not reached) with 2-year EFS of 78% (95% CI: 51–91). Three (18%) patients had a morphological relapse. The median RFS was not reached (IQR 47 months–not reached) with 2-year RFS of 82% (95% CI: 55–94). Seven patients died including three late deaths from hepatocellular carcinoma, pancreatic cancer and infection. The median OS was not reached (IQR 49 months–not reached) with 2-year OS of 77% (95% CI: 50–91). Of note, four out of five patients who were not transplanted did not relapse and the three patients with *NPM1* mutations were alive in CR1 at 3, 5 and 7 years. The two patients who had not received imatinib during induction relapsed and died. The comparison of de novo *BCR::ABL1*^+^AML, CML-BP, 2017 ELN intermediate (*n* = 643) or adverse-risk (*n* = 863) *BCR::ABL1* negative patients treated by intensive chemotherapy (DATAML registry) showed that patients with de novo *BCR::ABL1*^+^AML had a significant better outcome than 2017 ELN intermediate and adverse-risk patients (Table 3, Fig. 3). This result remained true after adjustment for age and allo-SCT in multivariate analyses (Supplementary Table 3).

**Table 3:** Outcome of patients with de novo *BCR::ABL1*^+^AML in comparison with CML-BP, ELN 2017 intermediate or adverse risk *BCR::ABL1* negative patients treated by intensive chemotherapy (DATAML registry).

	*De novo BCR::ABL1* AML *N* = 18	CML-BP *N* = 24	ELN 2017 intermediate *N* = 643	ELN 2017 adverse *N* = 863	*p*-value
CR/CRi (%)	94.4	79.2	83.4	68.5	<0.0001
Median OS (months)	NR	33.9	26.9	11.8	<0.0001
IQR	49–NR	15–NR	10–117	5–40	
2 year-OS (%)	77.4	57	54	33.4	<0.0001
95% CI	50–91	35–74	50–58	30–37	
5 year-OS (%)	62.5	42.2	34.5	20.8	<0.0001
95% CI	34–82	22–62	30–39	18–24	
Median EFS (months)	NR	29.7	15.5	7.2	<0.0001
IQR	49–NR	8–NR	5–72	3–23	
2 year-EFS (%)	77.8	57.6	40.8	21.5	<0.0001
95% CI	51–91	35–75	37–45	22–27	
5 year-EFS (%)	62.8	42.6	26.7	15.6	<0.0001
95% CI	34–82	22–62	23–30	13–18	
Median RFS (months)	NR	100.5	20.7	10.3	<0.0001
IQR	47–NR	20–NR	7–115	4–48	
2 year-RFS (%)	82.4	73.0	46.2	33.1	<0.0001
95% CI	55–94	47–88	42–50	29–37	
5 year-RFS (%)	66.6	54	31.5	22.1	<0.0001
95% CI	36–85	28–74	27–36	18–26	

*CML-BP* CML-blast phase, *ELN* European LeukemiaNet, *CR* complete remission, *CRi* complete remission with incomplete hematologic recovery, *IQR* interquartile range, *NR* not reached, *CI* confidence interval, *OS* overall survival, *EFS* event-free survival, *RFS* relapse-free survival.

## Discussion

In this study, we showed that de novo *BCR::ABL1*^+^AML represent a very rare entity of AML (0.3%) with particular features and outcome. Patients treated with intensive chemotherapy and imatinib have a very high rate of complete remission, very low incidence of relapse and a remarkable overall survival. Non-allografted patients may also have prolonged relapse-free and overall survival.

As no definite criteria exist to distinguish de novo *BCR::ABL1*^+^AML from CML-BP, we chose to adopt a pragmatic clinical definition of *BCR::ABL1*^+^AML (i.e., no previous history of CML, no previous treatment with TKI and ≥ 20% blasts in bone marrow) [3, 9]. Because 2022 ELN risk classification indicates that high-risk chromosomal abnormalities take precedence over *NPM1* mutations, we did not exclude patients who had both *NPM1* mutations and *BCR::ABL1*. With this definition, we confirmed previous reports showing that, compared with CML-BP, de novo *BCR::ABL1*^+^AML present with fewer additional chromosomal abnormalities, no *ABL1* mutations and a sizeable frequency of *NPM1* co-mutations [5, 7, 14, 15]. Moreover, we showed a higher number of mutations in de novo *BCR::ABL1*^+^AML although this was not statistically significant compared to CML-BP. We also demonstrated significant differences in immunophenotype distribution and in leukemic stem cell frequencies suggesting that the progression of CML to blast phase could be linked to the LMPP-like stem cells whereas de novo *BCR::ABL1*^+^AML may stem in other hematopoietic progenitor compartments. Additional studies with a higher number of cases are needed to confirm this preliminary data.

Of note, the female to male ratio was >1 in both CML-BP and de novo *BCR::ABL1*^+^AML, which is the opposite in both CML in chronic phase and AML. A recent study has also described sex-associated differences in frequencies of other genetic alterations in AML [16].

The most important result of our study is the efficacy and safety of imatinib in combination with standard intensive chemotherapy, which induced a very high rate of complete remission and no early death. Relapse-free and overall survival were better than in earlier studies that contributed to classify this entity as high-risk disease [4, 7, 14]. In a retrospective study from Korea on 29 patients with Ph+ AML including 7 with additional inv(16), the CR rate was 81.5% following imatinib added to intensive chemotherapy [17]. Two recent studies from transplantation registries showed a better outcome in de novo patients who received an allogeneic stem cell transplantation [18, 19]. In our study, the proportion of allografted patients (67%) may have contributed to the good outcome although the few non-transplanted patients did well too. Furthermore, the outcome of the three patients with *NPM1* mutations who were not transplanted was favorable. In a recent study demonstrating the adverse impact of high-risk cytogenetics in *NPM1* mutated patients, there were only two patients with t(9;22) [20]. Therefore, there is no clear evidence that *NPM1* mutated patients share the same unfavorable prognosis as patients with complex or monosomal karyotypes [21]. Those *NPM1* mutated patients should be preferably classified as favorable or intermediate risk according to *FLT3*-ITD mutation. On the other hand, this favorable outcome may also be related to the effectiveness of the addition of imatinib to the chemotherapy and not an inherently more favorable disease.

Regarding CML-BP, the CR/CRi rate was also favorable (79.2%) with a TKI added to the standard 3 + 7 and the 2 y OS of 57% compare favorably with a recent prospective study evaluating the more intensive FLAG-IDA regimen in combination with ponatinib in CML-BP of myeloid or lymphoid lineage [22].

Our study has shortcomings linked to its retrospective nature, the very low incidence of this disease imposing a very long study period, and some uncertainties concerning the distinction between de novo and CML-BP. Indeed, splenomegaly was not a criterion to distinguish de novo from CML-BP and basophil counts were not available in our AML database. Chemotherapy and imatinib doses were also somewhat heterogeneous during the study period. Due to the low number of patients, it is challenging to compare the results before and after TKI-era and a larger cohort of patients from international registries would be helpful for this purpose.

In conclusion, although further studies are needed to confirm these results and reclassify these AMLs in the intermediate or even favorable group of the 2022 ELN risk classification, our study showed that the combination of imatinib and standard intensive chemotherapy should be recommended in fit patients with de novo *BCR::ABL1*^*+*^ AML.

### Supplementary information

Supplementary table 1

Supplementary table 2

Supplementary table 3

Supplementary Figure 1 legend

Supplementary figure 1

## Data Availability

Requests for sharing deidentified data should be directed to the corresponding author.
