# Supplementary material for: Imatinib with intensive chemotherapy in AML with t(9;22)(q34.1;q11.2)/BCR::ABL1. A DATAML registry study
Source: Blood Cancer J. 2024 May 31;14(1):91. doi: 10.1038/s41408-024-01069-9 (PMC11143277; doi:10.1038/s41408-024-01069-9)
Supplement: Supplementary file 1 — Supplementary table 1 [file 41408_2024_1069_MOESM1_ESM.docx]

**Supplementary table 1: individual characteristics of patients with de novo *BCR::ABL1*^+^AML or CML-BP**

| **DATAML n°** | ***BCR::ABL1*^+^AML or CML-BP** | **Age/Sex** | **WBC (G/L)** | **EMD** | **Karyotype** | **% of Ph+ mitoses** | ***BCR::ABL1***  **fusion type** | **Mutations** | **IC** | **TKI** | **Response** | **AlloHCT** | **Relapse** | **Alive** |
| --- | --- | --- | --- | --- | --- | --- | --- | --- | --- | --- | --- | --- | --- | --- |
| 2000002 | *BCR::ABL1*^+^AML | 64/F | 35 | No | 46,XX,t(9;22)(q34;q11)[19]/46,XX[1] | 95% | Unknown | No NGS | Yes | No | CR | No | Yes | No |
| 2001050 | *BCR::ABL1*^+^AML | 47/M | 143 | SMG ADP | 47,XY,+6, t(9;22)(q34;q11)[18]/  48,sl, +7[1]/47, XY, +6[2] | 90% | P190 | *BCOR*  *NF1 RUNX1* | Yes | No | CR | Yes | Yes | No |
| 2005240 | *BCR::ABL1*^+^AML | 31/F | 14 | SMG | 46, XX, t(6;9;22)(p24;q34;q11)[15] | 100% | P210 | *IDH1 KDM6A* | Yes | Yes | CR | Yes | No | Yes |
| 2006277 | *BCR::ABL1*^+^AML | 22/M | 20 | No | 46,XY, t(9;22) (q34;q11)[10] | 100% | P210 | No NGS | Yes | Yes | CRi | Yes | Yes | No |
| 2009424 | *BCR::ABL1*^+^AML | 58/F | 5 | No | 46,XX,t(9;22)(q34;q11),inv(3)(q21q26)[20] | 100% | P210 | No NGS | Yes | Yes | CR | No | No | Yes |
| 20111053 | *BCR::ABL1*^+^AML | 50/F | 107 | No | 46,XX,t(9;22),(q34;q11)[12]/46,XX[1] | 92% | P210 | *ATM RUNX1 WT1* | Yes | Yes | CR | Yes | No | Yes |
| 20151723 | *BCR::ABL1*^+^AML | 56/M | 17 | HMG | 46,XY,t(1;2)(q41;q23),t(9;22)(q34;q11)[15]/46,XY[1] | 94% | P210 | *BCOR BCORL1 MPL, SMC1A STAG2 TET2* | Yes | Yes | CR | No | No | Yes |
| 20182315 | *BCR::ABL1*^+^AML | 70/F | 92 | HMG | 46,XX,t(9;22)(q34;q11)[20] | 100% | P210 | *EZH2 NPM1 RUNX1* | Yes | Yes | CR | No | No | Yes |
| 20150047 | *BCR::ABL1*^+^AML | 60/M | 90 | HMG  SMG ADP | 45,XY,t(9;22)(q34;q11),-16,der(17)t(16;17)  (q21;p12)[18]/46,sl,+der(17)t(16;17)[2] | 100% | P210 | None | Yes | Yes | CR | Yes | No | Yes |
| 20160050 | *BCR::ABL1*^+^AML | 29/M | 49 | No | 46,XY,t(9;22)(q34;q11)[20] | 100% | P210 | *NPM1* | Yes | Yes | CRi | Yes | No | Yes |
| 20190113 | *BCR::ABL1*^+^AML | 47/M | 372 | No | 46,XY,t(9;22)(q34;q11)[10] | 100% | P210 | None | Yes | Yes | CRi | Yes | No | Yes |
| 20180111 | *BCR::ABL1*^+^AML | 59/F | 99 | No | 46,XX,t(9;22)(q34;q11)[17]/46,XX[3] | 85 % | Unknown | *NPM1 TET2*  *WT1* | Yes | Yes | CRi | Yes | No | Yes |
| 20200138 | *BCR::ABL1*^+^AML | 45/F | 1 | HMG  SMG | 46,XX,t(9;22)(q34;q11)[6]/47,sl,+19[10]/  46,XX[4] | 80% | P210 | *ASXL1* | Yes | Yes | CRi | Yes | No | Yes |
| 20170084 | *BCR::ABL1*^+^AML | 51/F | 528 | No | 46,XX,t(1;3)(q31;q26),t(9;22)(q34;q11)[20] | 100% | P210 | *RUNX1* | Yes | Yes | CRi | Yes | Molecular | Yes |
| 20150020 | *BCR::ABL1*^+^AML | 69/M | 239 | SMG | 46,XY,t(9;22)(q34;q11)[25] | 100% | P210 | *ASXL1 DNMT3A* | Yes | Yes | CR | No | No | Yes |
| 20130067 | *BCR::ABL1*^+^AML | 59/M | 118 | No | 46,XY,t(9;22)(q34;q11)[24]/46,XY[1] | 96% | P210 | *ASXL1* | Yes | Yes | CR | Yes | No | No |
| 20150030 | *BCR::ABL1*^+^AML | 71/F | 149 | No | 46,XX,t(9;22)(q34;q11)[20]/46,XX[5] | 80% | P190 | *ASXL1 RUNX1 SRSF2* | Yes | Yes | Failure | No | No | Yes |
| 20172110 | *BCR::ABL1*^+^AML | 50/F | 21 | SMG | 46,XX,t(9;22)(q34;q11)[20] | 100% | P210 | None | Yes | Yes | CR | Yes | Molecular | Yes |
| 20170122 | *BCR::ABL1*^+^AML | 77/M | 26 | No | 46,XY,t(9;22)(q34;q11)[18]/46,sl,+1,dic(1;5)(p11;q15)[4]/46,XY[3] | 88% | P190 | *MYC ZSRS2* | No | Yes | CR | No | Yes | No |
| 20190199 | *BCR::ABL1*^+^AML | 63/F | 336 | SMG | 46,XX,t(9;22)(q34;q11)[20] | 100% | P210 | *ASXL1* | No | Yes | CRi | Yes | No | Yes |
| 2000634 | CML-BP | 66/F | 55 | SMG | 45,XX,-9,t(9;22)(q34;q11),add(17)(p11),  2min [17]/47,XX,sl,add(15)(q24)[3] | 100% | Unknown | No NGS | Yes | No | Failure | No | No | No |
| 2003119 | CML-BP | 39/F | 14 | SMG | 46,XX,t(9;22)(q34;q11)[1]/45,XX,sl,der(12;18)(q10;q10)[7]/45,XX,sdl,add(17)(q22)[2]/47,XX,sl,+der(22)t(9;22)[1]/46,XX,sl,der  (12;18),+der(22)t(9;22)[4]/46,XX,der(9)  t(9;22),ider(22)(10)t(9;22)[5] | 100% | Unknown | No NGS | Yes | Yes | CR | No | Yes | No |
| 2003130 | CML-BP | 52/M | 5 | No | 47,XY,t(7;19)(q22;p12),t(9;22)(q34;q11),  +21,var[17] | 100% | P210 | *RUNX1* | Yes | No | CR | Yes | No | Yes |
| 2004175 | CML-BP | 58/M | 222 | SMG | 43,XY,add(4)(q35),add(7)(p15),del(9)  (q11q21),t(9;22)(q34;q11),-12,der(14;17)  (q10;q10),del(16)(p12),-19[20] | 100% | Unknown | *TP53* | Yes | Yes | Death | No | No | No |
| 2004201 | CML-BP | 43/F | 5 | No | 46,XX,t(9;22)(q34;q11)[20] | 100% | P210 | No NGS | Yes | Yes | Failure | No | No | No |
| 2005239 | CML-BP | 65/F | 3 | No | 46,XX,t(9;22)(q34;q11)[4]/47,XX,sl,  +der(22)t(9;22)[10]/46,XX[6] | 70% | P190 | No NGS | Yes | Yes | CR | No | No | Yes |
| 2007327 | CML-BP | 52/F | 2,4 | No | 47,XX,t(2;7)(q26;p12),t(9;11)(p21;q21),  t(9;22)(q34;q11),add(16)(p11),del(17)(p12),+der(22)t(9;22) [22]/46,XX [8] | 73% | P210 | *BCOR* | Yes | No | CRi | Yes | Yes | No |
| 2007342 | CML-BP | 36/M | 124 | No | 46,XY,t(9;22)(q34;q11)[1]/46,sl,inv(3)  (q21q26)[17]/46,sdl,i(17)(q10)[2] | 100% | P210 | *ASXL1* | Yes | No | CRi | Yes | No | No |
| 20140103* | CML-BP | 51/M | 2,8 | No | 46,XY,del(5)(q31q34),del(7)(q21q31),-17,+mar[19]/46,XY[1] | 0% | NA | *No NGS* | Yes | No | CR | Yes | No | No |
| 20151635 | CML-BP | 66/M | 61 | No | 48,XY,der(2)t(2;22)(q33;q11),der(5)t(1;5)  (q12;q22),der(9)t(?8;9)(q22;q34),+21,  ider(22)(q10)t(9;22)(q34;q11),+der(22) add(22)(p11)t(9;22)[20] | 100% | P210 | *RUNX1 ABL1* | Yes | Yes | CR | Yes | No | Yes |
| 20151792 | CML-BP | 46/F | 31 | SMG | 46,XX,t(9;22)(q34;q11)[20] | 100% | P210 | *ASXL1 KMT2D* | Yes | Yes | CR | Yes | No | Yes |
| 20172124 | CML-BP | 64/F | 56 | No | 46,XX,t(9;22)(q34;q11)[20] | 100% | P210 | *RUNX1* | Yes | Yes | CR | No | No | Yes |
| 20172167 | CML-BP | 63/M | 215 | SMG | 46,XY,t(9;22)(q24;q11)[20] | 100% | P190 | *ASXL1 PHF6 RUNX1* | Yes | Yes | Death | No | No | No |
| 20172764 | CML-BP | 49/M | 237 | HMG  SMG ADP | 46,XY,t(9;22)(q34;q11)[1]/46,sl,t(1;3)  (p33;q26)[19] | 100% | P210 | None | Yes | Yes | CR | No | Yes | No |
| 20182417 | CML-BP | 42/F | 51 | SMG ADP | 46,XX,t(9;22)(q34;q11)[1]/46,sl,inv(16)  (p13q22)[16]/48,sdl, +marx2[3] | 100% | P210 | None | Yes | Yes | CRi | Yes | No | Yes |
| 20202844 | CML-BP | 29/F | 88 | HMG  SMG | 46,XX,t(9;22)(q34;q11)[22] | 100% | P210 | *ASXL1*  *IKZF1* | Yes | Yes | CRi | Yes | No | Yes |
| 20180019 | CML-BP | 43/M | 34 | HMG  SMG | 46,XY,t(9;22)(q34;q11)[13]/46,sl,t(8;17)  (q13;q23)[7] | 100% | Unknown | *WT1*  *ABL1* | Yes | Yes | CR | Yes | No | Yes |
| 20030086 | CML-BP | 29/F | 21 | HMG  SMG | 46, XX, add(4)(p11), add(8)(q22), t(9;22)(q34;q11)[35] | 100% | P210 | No NGS | Yes | Yes | CRi | Yes | No | Yes |
| 20170112 | CML-BP | 56/M | 3 | No | 46,XY,t(9;22)(q34;q11)[6]/46,XY[14].ish t(11;22)(q23;q1?3)[3].nuc ish (KMT2Ax2)  (5’KMT2A sep 3’KMT2Ax1)[16/100],  (MYH11,CBFB)X2[190/200] | 30% | P210 | *ASXL1* | Yes | Yes | CR | Yes | No | Yes |
| 20080085 | CML-BP | 54/F | 5 | No | 48,XX,t(5;9;22)(q23;q34;q11),+8,i(17)(q10),+der(22)t(9;22)[15]/46,XX[5] | 75% | P210 | No NGS | Yes | Yes | CR | Yes | Molecular | Yes |
| 20170120 | CML-BP | 57/M | 11 | No | 46,XY,t(3;21)(q26;q22),t(9;22)(q34;q11)[20] | 100% | P210 | None | Yes | Yes | CRi | Yes | Yes | No |
| 20120096 | CML-BP | 51/F | 97 | ADP | 46,XX,der(9)t(9;11;15)(p21;q23;q24),  t(9;22)(q34;q11),der(11)t(9;11;15),  der(15)t(9;11;15)[20] | 100% | P210 | *WT1* | Yes | Yes | CR | Yes | Yes | No |
| 20100089 | CML-BP | 51/M | 16 | Chloroma | 46,XY,t(9;22)(q34;q11)[8]/47,sl,+der(22)  t(9;22)[1]/48,sdl,+8,del(20)(q12)[2]/  46,XY[18] | 35% | P210 | None | Yes | Yes | CR | Yes | No | No |
| 20180110 | CML-BP | 52/F | 34 | SMG | 46,XX,t(2;10)(q21;q24),t(3;7)(q26;q21),  t(9;22)(q34;q11),del(12)(p11p13)[13]/  46,sl,inv(11)(p15q22)[7] | 100% | P210 | None | Yes | Yes | CRi | No | Molecular | No |
| 20111034 | CML-BP | 62/F | 1 | No | 45,XX,t(5;19)(p12;p12),-7,  t(9;22)(q34;q11)[10]/46,XX[2] | 83% | P210 | *ASXL1 KRAS SETBP1* | No | No | No | No | No | No |
| 20182330 | CML-BP | 56/F | 2 | No | 45,XX,inv(3)(q21q26),-7,  t(9;22)(q34;q11)[20] | 100% | P210 | None | No | Yes | Failure | No | No | No |
| 20000053 | CML-BP | 43/M | 8 | No | 46, XY, t(9;22;21) (q34;q11;q21) [23]/  47, sl,+ der(22)t(9;22;21)[11] | 100% | P210 | No NGS | No | Yes | Failure | No | No | No |
| 20180112 | CML-BP | 72/F | 38 | No | 45,XX,t(3;6)(q26;q26),-7,t(9;22)  (q34;q11)[20] | 100% | P190 | No NGS | No | Yes | Failure | No | No | No |
| 20220099 | CML-BP | 77/F | 4 | HMG | 46,XX,t(9;22)(q34;q11)[20] | 100% | P210 | No NGS - *ABL1 (E255K)* | No | Yes | Failure | No | No | No |

WBC, white blood cell count; EMD, extra medullary disease; IC, intensive chemotherapy; TKI, tyrosine kinase inhibitor; AlloHCT: allogeneic hematopoietic cell transplantation; CML-BP, CML blast phase; SMG, splenomegaly; ADP, adenopathy; HMG, hepatomegaly; CRi, complete remission (CR) with incomplete hematologic recovery.

* CML patient who presented with no Philadelphia chromosome at time of blastic progression.
