# Supplementary material for: Imatinib with intensive chemotherapy in AML with t(9;22)(q34.1;q11.2)/BCR::ABL1. A DATAML registry study
Source: Blood Cancer J. 2024 May 31;14(1):91. doi: 10.1038/s41408-024-01069-9 (PMC11143277; doi:10.1038/s41408-024-01069-9)
Supplement: Supplementary file 2 — Supplementary table 2 [file 41408_2024_1069_MOESM2_ESM.docx]

**Supplementary table 2: CML history in patients with CML-BP**

|  | **CML-BP (n=24)** |
| --- | --- |
| **Median time from CML diagnosis to blast phase, months, (IQR)** | 22 (7.2-104.6) |
| **Sokal score at CML diagnosis, n=14 (%)**  Low  Intermediate  High | 0  7 (50)  7 (50) |
| **ELTS score at CML diagnosis, n=9 (%)**  Low  Intermediate  High | 5 (55.6)  2 (22.2)  2 (22.2) |
| **First line treatment for CML*, n (%)**  Imatinib  Dasatinib  Nilotinib  Others (Hydroxyurea or Interferon) | 5 (20.8)  1 (4.2)  5 (20.8)  13 (54.2) |
| **Median duration of first line treatment, months, (IQR)** | 13.2 (6.1-104.5) |
| **Second line treatment for CML, n=11 (%)**  Imatinib  Dasatinib  Bosutinib  Ponatinib | 7 (63.6)  2 (18.2)  1 (9.1)  1 (9.1) |

CML, chronic myeloid leukemia; CML-BP, CML-blast phase; ELTS, EUTOS long-term survival score; IQR, interquartile range.* before blast phase.
