# Supplementary material for: Imatinib with intensive chemotherapy in AML with t(9;22)(q34.1;q11.2)/BCR::ABL1. A DATAML registry study
Source: Blood Cancer J. 2024 May 31;14(1):91. doi: 10.1038/s41408-024-01069-9 (PMC11143277; doi:10.1038/s41408-024-01069-9)
Supplement: Supplementary file 3 — Supplementary table 3 [file 41408_2024_1069_MOESM3_ESM.docx]

**Supplementary Table 3:** Multivariate analyses for complete remission, EFS, RFS and OS.

|  | **OR** | **95% CI** | ***P*** |
| --- | --- | --- | --- |
| ***CR/CRi*** | | | |
| *BCR::ABL1*^+^AML | 1 |  |  |
| CML-BP | 0.22 | 0.02-2.07 | 0.185 |
| 2017 ELN intermediate | 0.36 | 0.05-2.74 | 0.322 |
| 2017 ELN adverse (*BCR::ABL1*^-^) | 0.16 | 0.02-1.19 | 0.073 |
| Age>60y | 0.53 | 0.41-0.67 | <0.001 |
|  | **HR** | **95% CI** | ***P*** |
| ***Event-Free Survival*** | | | |
| *BCR::ABL1*^+^AML | 1 |  |  |
| CML-BP | 1.93 | 0.78-4.79 | 0.155 |
| 2017 ELN intermediate | 2.56 | 1.21-5.41 | 0.014 |
| 2017 ELN adverse (*BCR::ABL1*^-^) | 4.23 | 2.0-8.92 | <0.001 |
| Age>60y | 1.27 | 1.13-1.46 | <0.001 |
| Allo-SCT | 0.49 | 0.42-0.57 | <0.001 |
| ***Relapse-free Survival*** | | | |
| *BCR::ABL1*^+^AML | 1 |  |  |
| CML-BP | 1.57 | 0.56-4.40 | 0.396 |
| 2017 ELN intermediate | 2.61 | 1.16-5.85 | 0.02 |
| 2017 ELN adverse (*BCR::ABL1*^-^) | 4.05 | 1.81-9.10 | 0.001 |
| Age>60y | 1.15 | 0.99-1.33 | 0.055 |
| Allo-SCT | 0.49 | 0.42-0.58 | <0.001 |
| ***Overall Survival*** | | | |
| *BCR::ABL1*^+^AML | 1 |  |  |
| CML-BP | 1.92 | 0.78-4.77 | 0.158 |
| 2017 ELN intermediate | 2.02 | 0.95-4.27 | 0.066 |
| 2017 ELN adverse (*BCR::ABL1*^-^) | 3.65 | 1.73-7.70 | 0.001 |
| Age>60y | 1.28 | 1.13-1.46 | <0.001 |
| Allo-SCT | 0.47 | 0.40-0.55 | <0.001 |

*Allo-SCT: allogeneic stem cell transplantation (as a time dependent variable)

CR/CRi; complete remission (CR) or complete remission with incomplete hematologic recovery (CRi); OR, Odds-Ratio; HR, Hazard ratio.
