# Supplementary material for: Imatinib with intensive chemotherapy in AML with t(9;22)(q34.1;q11.2)/BCR::ABL1. A DATAML registry study
Source: Blood Cancer J. 2024 May 31;14(1):91. doi: 10.1038/s41408-024-01069-9 (PMC11143277; doi:10.1038/s41408-024-01069-9)
Supplement: Supplementary file 4 — Supplementary Figure 1 legend [file 41408_2024_1069_MOESM4_ESM.docx]

**Supplementary figure 1**

Volcano plot representing the proportion of the stage of leukemia arrest phenotypic subgroups expressed as odd ratios on the X-axis (positive values indicate a higher frequency) versus p values expressed on an inverted logarithmic scale on the y-axis. A. Comparison of CML-BP and de novo *BCR::ABL1* AML versus *BCR::ABL1* negative AML. B. de novo *BCR::ABL1* AML versus *BCR::ABL1* negative AML. C. CML-BP versus *BCR::ABL1* negative AML. MPP-L, multipotent progenitors-like; CMP-L, common myeloid progenitors-like; GMP-L, granulocyte–monocyte progenitors; GP-L, granulocyte progenitors-like ; MP-L, monocyte progenitors-like.
