# Supplementary figures and images for: Imatinib with intensive chemotherapy in AML with t(9;22)(q34.1;q11.2)/BCR::ABL1. A DATAML registry study
Source: Blood Cancer J. 2024 May 31;14(1):91. doi: 10.1038/s41408-024-01069-9 (PMC11143277; doi:10.1038/s41408-024-01069-9)

## Slide 1
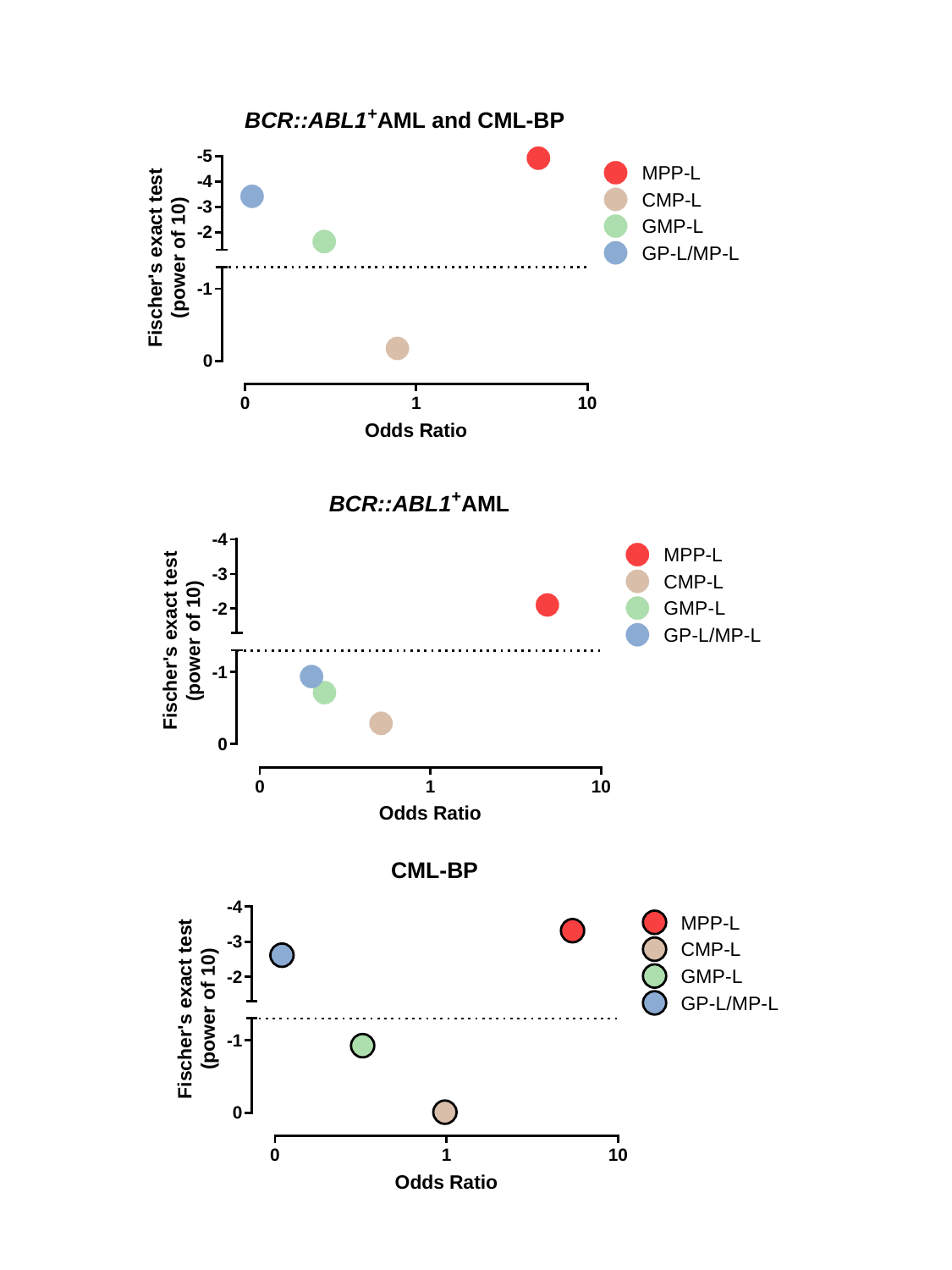

Supplement: Supplementary file 5 — Supplementary figure 1 [file 41408_2024_1069_MOESM5_ESM.pptx]
